# Non-invasive Loading Model of Murine Osteoarthritis

**DOI:** 10.1007/s11926-016-0590-z

**Published:** 2016-05-13

**Authors:** Blandine Poulet

**Affiliations:** Institute of Ageing and Chronic Disease, Musculoskeletal Biology 1, University of Liverpool, Room 286, Second Floor, Apex Building, West Derby Street, Liverpool, L7 8TX UK

**Keywords:** Osteoarthritis, Mouse model, Non-invasive, Mechanical loading

## Abstract

Osteoarthritis is the commonest degenerative joint disease, leading to joint pain and disability. The mouse has been the primary animal used for research, due to its size, relatively short lifespan, and the availability of genetically modified animals. Importantly, they show pathogenesis similar to osteoarthritis in humans. Mechanical loading is a major risk factor for osteoarthritis, and various mouse models have been developed to study the role and effects of mechanics on health and disease in various joints. This review describes the main mouse models used to non-invasively apply mechanical loads on joints. Most of the mouse models of osteoarthritis target the knee, including repetitive loading and joint injury such as ligament rupture, but a few studies have also characterised models for elbow, temporomandibular joint, and whole-body vibration spinal loading. These models are a great opportunity to dissect the influences of various types of mechanical input on joint health and disease.

## Introduction

Osteoarthritis (OA) is one of the commonest chronic diseases, affecting an estimated 9.6 % of men and 18 % of women over 60 years of age, which led the World Health Organization to classify OA as one of the ten most disabling diseases. Despite this high prevalence, there is currently no known therapy to prevent, slow, or repair joint degeneration. Severe cases often require surgical approaches, which include partial or total joint replacements. There are a number of risk factors, which include genetics, ageing, mechanical factors, and obesity. However, the exact aetiology of OA in individual patients is largely unknown and is most likely due to interactions among these risk factors.

Research into OA pathogenesis is necessary to define markers of disease and to define new targets for therapy. The use of human tissues in such research is of great benefit, but the lack of appropriate non-diseased controls at various ages and of tissues from early stages of disease makes the use of animal models necessary. In particular, the mouse has been accepted as a major tool for biomedical research. This is largely due to the ability to create a large number of genetically manipulated animals; their small size and short lifespan compared to larger mammals also allows them to be kept, bred, and aged at a relatively inexpensive cost and within an appropriate time frame for study design. The mouse is also an adequate model of human OA as it shares many of the hallmarks of human disease with many similarities in the mechanisms involved in the pathogenesis. Indeed, these hallmarks include articular cartilage (AC) degradation, subchondral bone sclerosis, osteophyte formation, and synovial inflammation and hyperplasia. Most OA research in mice has been done on knee joints, but a few models have also been developed for other joints, including elbow and temporomandibular joints and the spine.

Mechanical factors are well accepted as a major contributor to OA initiation and progression. Indeed, joint injury, participation in high-impact sports or highly repetitive manual labour has been correlated with increased risk of OA development [1–4]. At the same time, the lack of appropriate loading such as disuse or immobility can lead to cartilage thinning, decreased proteoglycan content, and OA development [5–7]. Animal models have also been used to show the role of mechanical disturbances, such as those seen with ligament transections and meniscectomies, on the promotion of OA development. The first post-traumatic OA animal model to be described was the Pond-Nuki model, which was performed in the dog by severing the anterior cruciate ligament (Pond-Nuki model; [8]). Subsequently, mouse models were developed in which different knee joint ligaments were transected, including the most common mouse model of OA used in OA research so far: the destabilisation of the medial meniscus (DMM). These models reproduce many of the characteristics of human OA, with signs of cartilage degradation from 2 weeks after surgery [9]. This is a reliable and reproducible technique to induce OA development in mouse knee joints. One major issue, however, is that it relies on surgery, which requires micro-surgical skills and may directly impact on disease progression due to disturbance of other periarticular tissues such as the fat pad. Recently, non-invasive mechanical loading models of OA have been developed [10] in various joints including knee, back, elbow, and temporomandibular joints.

## Non-invasive Mechanical Trauma

### Non-invasive Mechanical Load Application Murine Models

The effects of non-invasive repetitive loading were first investigated in the rabbit by Radin et al. [11]. In this model, the hindlimb was placed in a splint attached to a cam [11, 12] and subjected to 1.5 times the body weight for 40 cycles per minute for 7 or 20 days. An attenuated regime with an additional 4 weeks of rest was also developed by the same group to slow progression of OA-like changes. This model was less aggressive than the surgical models of OA. Recently, a few studies have used a similar system to apply quantifiable and controllable mechanical loads to the knee joint of a mouse. Most of these rely on similar approaches, which include the positioning of the mouse tibia between two custom cups at the level of the knee and the ankle [13••, 14••, 15, 16]. There are differences, however, among research laboratories, since the cups are custom-made with different specifications to the research groups. In addition, the protocols for applying the loads differ: loading may be initiated in the ankle cup [14••, 15] or in the knee cup [13••]. Other groups used lateral dynamic loads through the knee only [17]. In addition, the magnitude, frequency, cycle shape (i.e. trapezoidal, triangular, sinusoidal, or continuous displacement) and number, and number of days loaded vary dramatically among studies (Table 1). These will all contribute to differences in the severity of the tissue responses and make it difficult to compare results between groups. Nonetheless, all show relevant insights into the effects of mechanical loading on knee joint health and disease. Two main protocols are in use currently: repetitive loading and single injury.

**Table 1:** Summary of loading protocols for non-invasive murine knee joint loading models

Reference	Load direction	Magnitude	Cycle shape	Frequency	Load period	Rest period	Cycle number	Days of loading
Poulet et al. 2011 [12]	Knee→ankle	9 N	Trapezoidal	0.1 Hz	0.05 s	9.9 s	40	3 times/week
Ko et al. 2013 [15]	Ankle→knee	9 N	Triangular	4 Hz	0.125 s	0.125 s	1200	5 days /week
Wu et al. 2014 [14••]	Ankle→knee	3, 6, 9 N	Triangular	0.1 Hz	0.34 s	10 s	60	Once
Christiansen 2012 [13••]	Ankle→knee	12 N	Continuous displacement; 1 mm/s load rate				1	Once
Hamamura 2013 [16]	Lateral knee	3 N	Sinusoidal	5 Hz	0.2 s	0 s	1500	Once

### Repetitive Loading Models

Mouse knee repetitive loading models are based on studies of the effects of repetitive loading on bone architecture [18, 19]. The first one to be described by Poulet et al. [13••] used a magnitude (9 N) insufficient to induce osteogenic responses in the midshaft of the tibia [18], thus limiting the effects to the knee joint. Repetitive loading of 40 cycles six times over a period of 2 weeks induced localised articular cartilage lesions in the lateral femur. This study also showed that a single loading episode (40 cycles) was sufficient to induce cartilage lesions, but that these did not worsen with time alone. In contrast, 2 weeks of repetitive loading induced cartilage lesions that progressed with time and concurred with proteoglycan loss. This model therefore allowed differentiation between lesion induction and progression for the first time. This model was also used in the spontaneously osteoarthritic Str/ort mouse, which develops OA primarily in the medial tibia. Application of the same loading regime as that described above showed that Str/ort mice are in fact resistant to trauma-induced development of articular cartilage lesions in the lateral femur, and this feature was linked to the increased cartilage thickness in this strain [20]. In contrast, early spontaneous OA lesions on the medial tibia were accelerated by loading. Other joint tissues were also assessed in this model; indeed, repetitive mechanical loading led to localised subchondral bone and epiphyseal trabecular thickening at 5 weeks in the lateral tibia and femur [21]. These events are reminiscent of bone changes in human OA. Interestingly, epiphyseal trabecular mass was also increased across all four condyles in the contralateral non-loaded knee, which correlated with changes in gait in that leg. This supports the importance of using appropriate controls for such studies. Although pathological changes were seen in the knee joint ligaments in this model, including changes in matrix composition, cell shape, and cellularity, joint dislocation and ligament ruptures were not noted in this model.

The position of the tibia within the loading apparatus used by Poulet et al. [13••, 20] suggests that this model may also be useful to induce non-invasive trauma to the ankle joint. Indeed, the ankle joint is placed in the alignment of the loads being applied. This was tested in both CBA and C57Bl/6 mice that showed significant trauma in the knee joint loaded repetitively for 2 weeks (as described [13••, 21]), but no injury to the articular cartilage in the ankle has yet been found at 9 and 11 N (unpublished data). Further work is needed to define the appropriate loading regime that may lead to cartilage changes in the ankle joint trauma. This would be an important advance as there is currently only one known mouse model of ankle osteoarthritis, which relies on microsurgical transection of tendons and ligaments [22].

Another group used a repetitive regime over different periods of time, at higher frequencies, and 1200 cycles per day, for 5 days each week [16]. In this model, only the tibial plateau was studied and showed overt cartilage degeneration in the posterior compartment of the medial and lateral tibia. In addition, mechanical loading decreased bone mass in the epiphyseal trabecular bone, but increased bone mass in the metaphysis. Subchondral bone thickening was also seen in the posterior aspects of the joints, concurrent with the locations of articular cartilage lesions. However, these bone changes were compared to contralateral joints; although the authors state that the metaphysis does not show any contralateral changes in this model, the epiphysis was not tested. Thus, similar increases in bone mass, as seen in the model from Poulet et al. [21], might still be plausible. This study also showed the formation of osteophytes at the margins of the tibial plateau.

### Single-Impact Injury Models

Single-impact protocols have been used in similar systems, as described above. However, the loading protocols differ according to whether they induce joint dislocation and ligament ruptures. Indeed, Christiansen et al. [14••] applied continuous loading to reach the point of rupture of the cruciate ligaments. This injurious loading resulted in cartilage degradation with moderate to severe injury, knee dislocation, and avulsion fractures that are consistent with anterior cruciate ligament (ACL) ruptures and initial bone loss. Modification in the speed of loading was shown to lead to ACL disruption without avulsion fractures; however; no difference in severity of progressing OA was seen between high and low loading rates [23]. At both loading rates, severe degeneration was seen at 12 and 16 weeks after injury in all four quadrants of the knee joint, with very severe erosion of the subchondral plate to the tidemark, in some instances. There was also extreme fibrosis within the joint space and formation of osteophytes, as well as hypertrophied and degenerated menisci. This severe model of OA was used to determine enzyme activity in vivo, using fluorescent reflectance imaging [24]; this showed that, at early time points following injury, proteases, matrix metalloproteinases, and cathepsin K activities were increased in injured joints.

Another group used a single loading episode, composed of 60 cycles of 9 N peak loads for 0.34 s, interspersed with 10 s of rest time, to induce joint translation and ACL rupture [15]. Articular cartilage lesions were seen alongside proteoglycan loss and TUNEL-positive apoptotic cells. Immunostaining for matrix proteins in the injured cartilage showed decreased pericellular aggrecan thickness and intensity and increased abnormal traces of collagen type I, but no visible change in the cartilage-specific collagen type II. Signs of synovitis were also seen in the injured knee from 5 days after the loading with increased synovial cell proliferation and lining cell hyperplasia. Signs of neocartilage tissue appeared in the synovium and meniscus.

Ko et al. [25] studied the effects of a single session of their protocol (consisting of 9 N loads, at 4 Hz for 1200 cycles) that lead to significant cartilage lesions after 1 week of the tibia (again, the femur was not assessed). Epiphyseal bone mass was transiently decreased after 1 week and came back to normal at 2 weeks, whereas the subchondral bone plate thinning was enhanced between 1 and 2 weeks. In addition, no synovial inflammation was detected in these joints. This protocol seems to promote cartilage damage, according to the authors without anterior cruciate ligament injury, and with bone changes that partially resolve, although the long-term effects on OA development and progression have not been assessed in this study.

These single-impact injury models are more closely related to the surgical models of ligament transection in that they induce permanent mechanical disturbances and severe fast OA development, as opposed to the transient nature of the repetitive loading regimes without ligament rupture described above leading to milder slow OA development. Compared to commonly used surgical models of OA, however, these in vivo models of applied mechanical loading have the advantage of using quantifiable forces to the joints. Single-impact models, however, are mainly applicable to ligament injury-induced OA. Repetitive loading could be seen as more representative of day-to-day activities that might lead to OA development. But it remains difficult to compare all of these non-invasive models because of the variations in load applications, protocols, and time points used. A consensus on some of these parameters needs to be discussed and agreed upon. Nevertheless, some clear similarities in the pathogenesis of OA generated with all of these models suggest that common pathways may be in play and remain important for the definition of targets for posttraumatic OA.

## Exercise Model

Exercise, in particular at the elite level, has been linked to increased risk of OA [26, 27]. However, this remains controversial with some studies not finding any significant changes in professional runners [28–30]. Nevertheless, it is pertinent to use exercise as a modulator of mechanical loads in weight-bearing joints such as the knee. Rodents can be trained to run on wheels voluntarily throughout life. This approach has been used to show that transgenic mice susceptible to OA development, such as Del1 mice which harbour a mutated collagen type II gene, develop more severe disease with running [31]. Mice can also be trained to use a treadmill for more controlled running exercise with a preselected speed and length of the running episode. Thus, C57BL6 mice which develop OA spontaneously by 18 months of age, showed increased incidence of severe OA in both lateral and medial tibiae, especially after 16 months of running (from 2 months of age to 18 months), for 75 min a day for 5 days a week [32]. However, it seems that specific regimes and time may be required, as 2 months of treadmill exercise for 30 min/day and 5 days/week for 8 weeks was not sufficient to induce any histological changes in joints of mice without or with deletion of superoxide dismutase-2, although chondrocytes and bone cells did show cellular responses to the exercise [33].

Although long periods of time are needed to see the effects on joints, exercise has the advantage of directly representing a specific type of OA patient (i.e. elite runners). Alternatively, exercise can be used in conjunction with other OA models, such as genetic or surgical models, to create a more severe disease.

## Mechanical Loading of Other Joints

OA affects many joints in the body. However, the majority of the research concentrates on the knee joint, which is the most affected in human patients. Some models have recently been developed and described for loading of the elbow, the temporomandibular joint (TMJ), and intervertebral discs.

### Elbow Loading

Elbow OA is primarily due to mechanical injury [34]. Mechanical loading of the ulna in mice has been used previously to study the effects of mechanical input on bone physiology [35]. Thus, one group has used this model to determine the effects of non-invasive loading of the elbow joint on articular cartilage enzyme gene expression [36]. They found that 1 h after the load application, low magnitudes (0.2, 0.5 N) reduced metalloproteinase (MMP) gene expression and collagenase activity, whereas high loads (2 N) increased gene expression of MMPs and their inhibitors TIMPs. The authors did not, however, analyse morphological changes within the joint such as cartilage degeneration, and thus, it is currently unclear whether this can be used as a model of elbow OA.

### Temporomandibular Joint Loading

Temporomandibular joint (TMJ) pain affects 10 % of the population, and 15 % of these show signs of TMJ degeneration [37]. Three non-invasive models have been used in the mouse. The first two rely on the animals chewing on pellets of different softness (normal hard pellet versus soft dough; [38]), but with similar nutritional values, with/without incisor trimming every other day to alter mechanical loading. They found that articular cartilage gene expression was modified in the altered mechanical loading group compared to normal loading (hard pellet), with decreased articular cartilage thickness and collagen type II immunolabelling. In addition, this study did not show any significant degeneration in the joint during the 6 weeks of the experiment. Similarly, mastication can be modified to alter mechanical loads in the TMJ. Liu et al. [39] reduced mastication by reducing the size of the pellets, as well as adding a unilateral anterior crossbite prosthesis that increased the demands on the TMJ during mastication. After 3 weeks, cartilage thinning, loss of proteoglycan and collagen type II, and decreased cellularity were noted in the TMJ of the prosthesis group chewing on large pellets and, to a lesser extent, in the prosthesis group chewing small pellets. Although these models can be used without any significant input from the lab technician and normal behaviour can be restored, it is not easy to control the loading being applied. In addition, using pellet softness and size alone are not sufficient to induce any significant degenerative changes.

The third model that has been developed for TMJ loading is more invasive, as it relies on continuous, forced-mouth opening using a spring while under anaesthesia for 1 h/day for 5 days [40••]. But this allowed for specific forces to be applied and showed increased chondrocyte proliferation, increases in gene expression of cartilage anabolic markers Sox9 and collagen type II, and subchondral bone thickening. This was then used in transgenic mice to show increased cartilage thickness in young growing mice, with increased remodelling markers to meet changes in mechanical forces [41].

Although these models have been successfully used to determine the effects of altered mechanical loads on the TMJ, they have not yet been tested for TMJ degeneration, and further experiments of longer duration and with older mice may be needed to test their relevance to disease.

### Intervertebral Disc Degeneration

Back pain and degeneration are major contributors to disability in the human population [42]. The aetiology of intervertebral disc degeneration remains elusive, and research into the roles of mechanical loading and trauma as the main causes of disease has involved mainly in vitro studies. Some in vivo models have now been developed and used, although most rely on genetic susceptibility (specific dog breeds; [43]) or invasive procedures to induce injury to the discs (such as punctures; [44, 45]). Recently, a non-invasive model of mechanical loading has been tested in mice, where a vibration platform is used, similar to those used in humans to improve bone quality. McCann et al. [46, 47••] used a custom-made apparatus for whole-body vibration in mice, using a loading protocol similar to that used in human clinics. They showed that single episodes, lasting 30 min, induced increased anabolism in the intervertebral disc with increased matrix proteins (aggrecan, biglycan, decorin) and decreased degradative enzymes (MMP3, ADAMTS4/5; [46]), and may therefore be beneficial. In contrast, repetitive vibration for 4 weeks induced degeneration [47••], with increased enzyme expression and activity. This suggests that repetitive whole-body vibrations in mouse could be used as a non-invasive model of loading of the back leading to intervertebral disc degeneration.

## Conclusions

There has recently been a description of models for non-invasive mechanical loading in the mouse to study OA development, involving repetitive mild loading regimes, single traumatic events that induce ligament ruptures, and exercise regimes for knee osteoarthritis. In addition, few groups have attempted to develop models for other joints in the mouse including the elbow, the TMJ, and the back, all of which represent important patient subsets. The main advantage of these models is their non-invasive nature, where no specific microsurgical skills are needed, making this model theoretically more reproducible between groups, as well as reducing the potential effects of surgery on disease development. In addition, some of these models that do not induce ligament ruptures have the ability to be transient in nature, as opposed to inducing permanent mechanical disturbances in the more severe models. These models have not yet, however, been tested to define specific markers or targets for OA therapy. Further work using these models will significantly add to the current research being done on surgical and spontaneous models of OA and represent a great opportunity to dissect the influence of various types of mechanical input on joint health and disease.
